# Coordinated Expression of HPV-6 Genes with Predominant E4 and E5 Expression in Laryngeal Papilloma

**DOI:** 10.3390/microorganisms9030520

**Published:** 2021-03-03

**Authors:** Taro Ikegami, Hitoshi Hirakawa, Narutoshi Tsukahara, Akikazu Murakami, Norimoto Kise, Asanori Kiyuna, Takayoshi Kosugi, Shinya Agena, Hidetoshi Kinjyo, Narumi Hasegawa, Masatomo Touyama, Shunsuke Kondo, Hiroyuki Maeda, Mikio Suzuki, Akira Ganaha

**Affiliations:** 1 Department of Otorhinolaryngology, Head and Neck Surgery, Graduate School of Medicine, University of the Ryukyus, Okinawa 903-0215, Japan; ikegami@med.u-ryukyu.ac.jp (T.I.); hanntagawa@hotmail.com (H.H.); norimoto7@gmail.com (N.K.); jibika_asanori97@yahoo.co.jp (A.K.); tnq5@wildcats.unh.edu (T.K.); harugen3@yahoo.co.jp (S.A.); hidechanman223@yahoo.co.jp (H.K.); br101426@gmail.com (N.H.); puyoraer99110@gmail.com (M.T.); kouhouiinn@yahoo.co.jp (S.K.); maeidahiroyuki@yahoo.co.jp (H.M.); ganaha.akira.t8@cc.miyazaki-u.ac.jp (A.G.); 2 Department of Parasitology & Immunopathoetiology, Graduate School of Medicine, University of the Ryukyus, Okinawa 903-0215, Japan; tsukahara@rephagen.com (N.T.); akimu@med.u-ryukyu.ac.jp (A.M.); 3 RePHAGEN Co., Ltd., Okinawa 904-2234, Japan; 4 Department of Otolaryngology, Faculty of Medicine, University of Miyazaki, Miyazaki, Miyazaki 889-1692, Japan

**Keywords:** laryngeal papilloma, human papillomavirus 6, viral lineage, viral mRNA expression, long-term alteration of viral load, anti-E4 monoclonal antibody

## Abstract

Laryngeal papilloma (LP) associated with human papillomavirus (HPV)-6 or -11 infection shows aggressive growth. However, the detailed molecular mechanism of virus-driven tumorigenesis has not been uncovered fully. HPV-6 viral gene expression and dynamic alterations were investigated with in situ localization of viral DNA and RNA in 13 patients with HPV-6-infected laryngeal papilloma. The average viral load was 4.80 × 10^5^ ± 1.86 × 10^5^ copies/ng DNA. *E4*, *E5a*, and *E5b* mRNAs accounted for 96% of the expression of 9 mRNAs. The alteration of viral DNA load during recurrence paralleled the mRNA expression levels, and the expression of all mRNAs showed a similar curve. *E4*, *E5a*, and *E5b* were expressed in the middle to upper part of the epithelium and were co-expressed in the same cells. E4 immunohistochemistry demonstrated an extensively positive reaction in the upper cell layer in accordance with *E4* mRNA expression. These results suggest that individual viral genes are coordinately expressed for viral replication, virus release, and immunosurveillance avoidance. The newly developed E4-specific monoclonal antibody can be applied to further functional studies and clinical applications such as targeted molecular therapies.

## 1. Introduction

Laryngeal papilloma (LP) is the most common benign epithelial tumor of the larynx, appearing as finger-like projections or multiple fronds of stratified squamous epithelium. The typical pathological findings are hyperplasia of basal cells and large vacuolated epithelial cells with clear cytoplasm. The estimated incidence of LP is approximately 4 per 100,000 in children and 2 per 100,000 in adults [1]. LP can form multiple tumors and has a high recurrence rate. Moreover, LP in children sometimes shows aggressive growth with rapid recurrence and tracheobronchial extension [2], although the malignant transformation of LP has been reported in less than 3% of cases [3,4]. Frequent surgery and occasional tracheostomies are needed to maintain the airway in some cases [2]. Since human papillomavirus (HPV)-6 and -11 infections are usually detected in LP, these viral infections are thought to be a distinct etiology of LP.

HPVs are small double-stranded DNA viruses that infect mucosal and cutaneous epithelia and cause many tumorous lesions from benign warts to cancer of the anogenital and otolaryngological regions [5]. The approximately 8000 bp HPV genome is divided into 3 regions, i.e., 6 early regions (*E1*, *E2*, *E4*, *E5*, *E6,* and *E7*), 2 late regions (*L1* and *L2*), and the long control region (LCR). According to their association with cancer, HPV is divided into low-risk and high-risk types. HPV-6 and -11, observed in LP, are classified as the low-risk type. HPV preferentially infects mitotically active basal cells through microtears in the mucosal and cutaneous epithelium [6]. The viral life cycle synchronizes with the epithelial differentiation program, which may be due to the binding of differentially expressed cellular transcription factors to the LCR throughout the various epithelial layers [7]. Despite variation in the size and number of open reading frames, all papillomaviruses contain well-conserved core genes involved in replication (*E1* and *E2*) and packaging (*L1* and *L2*) with greater diversity in the remaining genes (*E6*, *E7*, *E5*, and *E4*), which have roles in driving cell cycle entry, immune evasion, and virus release [8]. Although the expression of these viral genes and their roles have been investigated extensively in high-risk HPVs [6,8], they remain unclear in LP with low-risk HPV infection, such as the cells that express these genes and the dynamic variations of HPV viral load in primary tumors and recurrent tumors after treatment [9]. 

**Figure 1:**
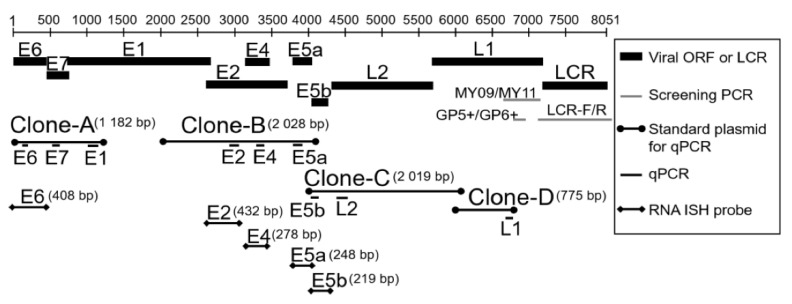
Schema of human papillomavirus-6 (HPV-6) genes and information of plasmid clones and RNA in situ hybridization probes used in this study.

*E4* is located centrally within the *E2* gene (Figure 1), and the primary *E4* gene product (*E1^E4*) is translated from a spliced mRNA [10]. Although several minor *E4* transcripts have been reported, it is the product of the abundant *E1^E4* mRNA that has been most extensively analyzed [11]. *E4* protein encoded by HPV-16 appears to have the ability to bind to keratin and to reorganize the cellular keratin network, suggesting a role in virus release and transmission [10]. HPV-6 and -11 contain a second open reading frame downstream of *E5a*, which is called *E5b*. *E5b* shows no sequence similarity to other *E5* proteins. Although HPV-16 *E5* can activate the epidermal growth factor receptor, inhibit the expression of the p21 tumor suppressor gene [12,13], and has a role in immunoevasion [14], the biological functions of *E5a* and *E5b* in low-risk HPVs are not understood fully. 

LP caused by HPV-6 and -11 infection is a chronic and recurrent disease that significantly lowers patients’ quality of life and may lead to life-threatening conditions. Furthermore, effective clinical treatments have not been established. Therefore, it is vitally important to characterize the underlying pathogenic mechanisms. However, the detailed molecular mechanisms of HPV-driven tumorigenesis are mostly unknown. In the present study, we established a real-time polymerase chain reaction (PCR) assay to measure the absolute levels of *E6*, *E7*, *E1*, *E2*, *E4*, *E5a*, *E5b*, *L2*, and *L1* mRNAs, an mRNA in situ hybridization (ISH) method for *E6*, *E2*, *E4*, *E5a*, and *E5b*, and created an anti-E4 monoclonal antibody for immunohistochemical localization of E4 in LP.

## 2. Materials and Methods

### 2.1. Subjects

The subjects consisted of 16 patients with LP aged from 4 to 67 years at the first visit to our hospital between 2010 and 2018. Tumor specimens were frozen immediately in liquid nitrogen at biopsy or surgical excision between 2010 and 2018 and stored at −80 °C until analysis. 

The HPV subtypes were determined as described previously [15]. Of the 16 patients, 13 (81.3%) had HPV-6 and 3 (18.7%) had HPV-11. Since the major HPV subtype was HPV-6, the present study focused on HPV-6-infected LP and analyzed the viral DNA load, mRNA expression of viral genes, and cellular localization of viral genes and proteins by PCR, real-time PCR, DNA- and RNA-ISH (in situ hybridization), and immunohistochemistry using self-made probes and antibodies. Since sufficient fresh-frozen samples were obtained from 10 of 13 LP patients, mRNA expression was evaluated using these samples (Table 1). Several fresh-frozen samples at sites of recurrence were obtained from 2 of 13 patients with HPV-6-positive LP (cases 9 and 10).

### 2.2. Detection of Human Papillomavirus (HPV) DNA and Quantitative Polymerase Chain Reaction (PCR) Analysis of HPV-6 Viral Load

#### 2.2.1. Extraction of Genomic DNA from Laryngeal Papilloma (LP) and PCR Conditions

DNA was extracted from fresh-frozen samples using a Gentra Puregene Tissue Kit (QIAGEN, Gaithersburg, MD, USA) [15]. The presence and integrity of DNA in all samples were investigated by PCR amplification of the β-globin gene using primers PC04 and GH20. The primers used in the present study are shown in Table S1 in the Supplementary Materials. The amplification efficiency of target genes is shown in Table S2 in the Supplementary Materials.

Negative (water) and positive (DNA from an HPV-16-positive CaSki cell line) controls were included in each amplification series. To determine the HPV subtypes in the samples, we used the degenerate consensus primer sets GP5+/GP6+ and MY09/MY11 [16], which were designed to amplify the *L1* region. When no PCR amplification occurred with the GP5+/GP6+ or MY09/MY11 primers, 10-fold diluted first-round PCR products were used as template DNA for nested PCR using the GP5+/GP6+ primer pair. PCR products of the expected size (GP5+/GP6+, 150 bp; MY09/MY11, 450 bp) were purified and directly sequenced with an ABI PRISM 3130xl Genetic Analyzer (Applied Biosystems; Thermo Fisher Scientific, Inc., Waltham, MA, USA). The sequences were aligned and compared with the *L1* gene of known HPV subtypes in GenBank using blastn: https://blast.ncbi.nlm.nih.gov/Blast.cgi?PROGRAM=blastn&PAGE_TYPE=BlastSearch&LINK_LOC=blasthome (accessed on 13 February 2021).

#### 2.2.2. Partial Cloning of the HPV-6-LCR Region and Identification of HPV-6 Subtypes

To identify the HPV-6 subtype, we designed LCR-degenerate primers in the conserved region from *L1* to E6 of HPV-6a (GenBank accession no. L41216), HPV-6b (X00203), and HPV-6vc (JN252316). For amplification, PCR was performed with LCR-F and LCR-R primers (0.24 μM each, Table S1 in the Supplementary Materials) in a mixture (12.5 μL) containing 6.3 μL GoTaq^®^ Green Master Mix (Promega, Madison, WI, USA) and 10 ng genomic DNA. The PCR profile was as follows: denaturation at 95 °C for 15 min, followed by 45 cycles at 95 °C for 15 s, 60 °C for 30 s, and 72 °C for 2 min, and a final extension at 72 °C for 5 min. PCR fragments of the expected size were purified by the Wizard SV Gel and PCR Clean-Up System (Promega, Madison, WI, USA). The purified PCR products were cloned into the pGEM-T Easy vector (Promega, Madison, WI, USA) and sequenced with an ABI PRISM 3130xl Genetic Analyzer (Applied Biosystems, Foster City, CA, USA). 

The obtained nucleotide sequences of the LCR region were compared with HPV-6a, HPV-6b, and HPV-6vc by the GENETYX sequence analysis software package (Software Development Co., Ltd., Tokyo, Japan). Multiple alignments for phylogenetic analysis were performed by the GENETYX sequence analysis software package. A phylogenetic tree was constructed by the unweighted pair group method with arithmetic mean, using the GENETYX sequence analysis software package. The LCR region sequence of HPV-11 served as an outgroup for the phylogenetic tree.

#### 2.2.3. Quantitative PCR Analysis of HPV-6 Viral Load in LP

To evaluate the viral load of HPV-6-infected LP, quantitative PCR was performed as described previously [17]. HPV-6 *E6* DNA copy number in genomic DNA was determined with the ABI Prism 7300 Sequence Detection System (Applied Biosystems, Foster City, CA, USA) or CFX96 Touch™ Real-Time PCR Detection System (Bio-Rad, Hercules, CA, USA). Briefly, serially diluted HPV-6 *E6* plasmid (p1478 HPV-6 *E6*; Addgene, Cambridge, MA, USA) was used as a standard (1.0 × 10^1^–1.0 × 10^7^ copies/2 μL) to establish a standard curve. p1478 HPV-6 *E6* was a gift from Peter Howley (Addgene plasmid #10872; http://n2t.net/addgene:10872; accessed on 13 February 2021). The genomic DNA samples from the patients were diluted to 30 ng/2 μL and used for measurement. The PCR reaction mixture (10 μL) contained 0.2 μM primers, 0.15 μM TaqMan probe (Table S1 in the Supplementary Materials), 5 μL TaqMan Universal Master Mix II with Uracil-DNA Glycosylase (UNG; Applied Biosystems, Carlsbad, CA, USA), and 2 μL template DNA. The PCR profile was as follows: 50 °C for 2 min and 95 °C for 10 min, followed by 40 cycles at 95 °C for 15 s and 60 °C for 1 min. To quantify sample DNA amounts, an external standard curve was created using known serial dilutions (0.3, 3, 30, and 300 ng) of human placental genomic DNA (Sigma-Aldrich, Merck KGaA, Darmstadt, Germany), and β-globin was amplified as described previously [17]. Viral load was defined by *E6* copy number/ng cellular DNA.

### 2.3. Measurement of Viral mRNA Expression in HPV-6-infected LP by Quantitative Real-Time PCR

According to the manufacturer’s instructions, total RNA was extracted from fresh-frozen LP samples with RNAiso Plus (Takara, Otsu, Japan). Total RNA (500 ng) from each sample was reverse-transcribed using the PrimeScript^R^ RT Reagent with gDNA Eraser (Takara, Otsu, Japan). To establish real-time PCR assays to measure the absolute levels of *E7*, *E1*, *E2*, *E4*, *E5a*, *E5b*, *L2,* and *L1* mRNAs, we cloned 4 regions of HPV-6 gene fragments using genomic DNA from patient 3 (clones A, B, C, and D in Figure 1). Clone A was amplified from the anterior region of the *E6* gene to the anterior one-third of the *E1* gene (nt5–nt1186, 1182 bp) using primer pairs (*F1* and *R1* in Table S1 in the Supplementary Materials). Clone B was amplified from the posterior one-third of the *E1* gene to the anterior one-half of the *E5b* gene (nt2117–nt4144, 2028 bp) using primer pairs (*F2* and *R2* in Table S1 in the Supplementary Materials). Clone C was amplified from the anterior region of the *E5b* gene to the anterior one-third of the *L1* gene (nt4005–nt6023, 2019 bp) using primer pairs (*F3* and *R3* in Table S1 in the Supplementary Materials). Clone D was amplified from the anterior one-third of the *L1* gene to the posterior one-third of the *L1* gene (nt6001–nt6775, 775 bp) using primer pairs (*F4* and *R4* in Table S1 in the Supplementary Materials). The PCR reaction mixture (12.5 μL) contained 1 μL genomic DNA from patient-3 (30 ng/μL), 0.24 μM forward and reverse primers, and 6.3 μL GoTaq^®^ Green Master Mix (Promega, Madison, WI, USA). The PCR conditions comprised: 95 °C for 15 min, followed by 40 cycles at 95 °C for 30 s, 55 °C for 30 s, and 72 °C for 3 min, and finally 72 °C for 7 min. The PCR fragments of the expected size were purified using the Wizard SV Gel and PCR Clean-Up System (Promega, Madison, WI, USA). The purified PCR products were cloned into the pGEM-T Easy vector (Promega, Madison, WI, USA) and sequenced using an ABI PRISM 3130xl Genetic Analyzer. The sequences obtained were analyzed as mentioned above. The plasmids (clones A, B, C, and D) were serially diluted to 2.0 × 10^1^–2.0 × 10^7^ copies/2 μL as standards for *E7*, *E1*, *E2*, *E4*, *E5a*, *E5b*, *L2,* and *L1* genes to establish a standard curve in real-time PCR. Real-time PCR was performed with the ABI Prism 7300 Sequence Detection System (Applied Biosystems, Foster City, CA, USA) or CFX96 Touch™ Real-Time PCR Detection System (Bio-Rad, Hercules, CA, USA). The PCR reaction mixture (10 μL) contained 0.2 μM primers, 5 μL SYBR Premix Ex Taq^TM^ II (Tli RNaseH plus; Takara, Otsu, Japan), and 2 μL standard plasmid DNA (2.0 × 10^1^–2.0 × 10^7^ copies) or cDNA. The PCR profile was as follows: 95 °C for 30 s followed by 40 cycles at 95 °C for 5 s and 60 °C for 30 s. Specific amplification of each cDNA was verified by melting curve analysis and gel electrophoresis of the products. The real-time PCR assay for *E6* mRNA was performed with the same *E6* primers and TaqMan probe as well as the same protocol described above. We also measured β-actin mRNA as the internal reference. The plasmid pCAG-mGFP-Actin (a gift from Ryohei Yasuda), carrying the entire coding region of β-actin (Addgene plasmid #21948; Addgene, Cambridge, MA, USA), was serially diluted to 1.0 × 10^1^–1.0 × 10^7^ copies/2 μL to establish a standard curve. The PCR reaction mixture (10 μL) contained 0.2 μM primers, 0.15 μM TaqMan probe (Table S1 in the Supplementary Materials), 5 μL TaqMan Universal Master Mix II with UNG, and 2 μL template DNA. The PCR profile was as follows: 50 °C for 2 min and 95 °C for 10 min, followed by 40 cycles at 95 °C for 15 s and 60 °C for 1 min. β-Actin mRNA levels were used to normalize all viral mRNA levels. 

### 2.4. In Situ Hybridization (ISH) with HPV DNA Probes

Biotinyl tyramide-based ISH was performed using the HPV Types 6/11 Biotinylated DNA Probe (Y1411; Dako, Agilent Technologies, Inc., Santa Clara, CA, USA) and the GenPoint tyramide signal amplification system for biotinylated probes according to the manufacturer’s protocol (Dako, Agilent Technologies, Inc., Santa Clara, CA, USA). The biotinylated DNA probe has been found to react with HPV-6 and -11 in formalin-fixed paraffin-embedded (FFPE) sections by ISH. Serial 4-µm-thick sections of FFPE samples were deparaffinized in xylene and rehydrated using a graded alcohol series. Target HPV DNA retrieval was performed in 10 mM sodium citrate (at pH 6.0) at 95 °C for 40 min followed by a 20-min cooldown period. The slides were digested with proteinase K (10,000 times dilution with Tris-buffered saline; Dako, Agilent Technologies, Santa Clara, CA, USA) for 10 min at room temperature. Endogenous peroxidases were blocked with 0.3% H_2_O_2_ in methanol for 20 min. A drop of the HPV probe was added to the section, and a coverslip was applied. The probe and target DNA were denatured by incubating the slides at 92 °C for 5 min. After denaturation, the slides were transferred to a humidified chamber for hybridization at 37 °C for 16 h. After hybridization, the coverslips were removed, and the slides were bathed in Tris-buffered saline containing 0.05% Tween 20 (TBST). The coverslips were washed using GenPoint Detection system stringent wash solution (Dako, Agilent Technologies, Santa Clara, CA, USA) at 48 °C for 30 min, following by rinsing in TBST. Detection of the hybridized probe was performed using the GenPoint Detection system with primary streptavidin-horseradish peroxidase (HRP), biotinyl tyramide, secondary streptavidin-HRP, and 3-3′-diaminobenzidine (Dako, Agilent Technologies, Santa Clara, CA, USA). The slides were counterstained with hematoxylin.

### 2.5. RNA-ISH with HPV-6 E6, E2, E4, E5a, and E5b Digoxigenin RNA Probes

For RNA-ISH, the *E6*, *E2*, *E4*, *E5a*, and *E5b* genes of HPV-6 were amplified by the following PCR method. The primer sets are shown in Table S1 in the Supplementary Materials. The PCR reaction mixture (12.5 μL) contained 1 μL template plasmid (clone A plasmid for *E6*; clone B plasmid for *E2*, *E4*, and *E5a*; clone C plasmid for *E5b*), 0.24 μM forward and reverse primers, and 6.3 μL GoTaq^®^ Green Master Mix (Promega, Madison, WI, USA). The PCR conditions comprised: 95 °C for 5 min, followed by 35 cycles at 95 °C for 30 s, 60 °C for 30 s, and 72 °C for 1 min, and finally 72 °C for 5 min. The PCR products were subcloned into the pGEM-T Easy vector (Promega, Madison, WI, USA) as described above. Anti-sense RNA probes were transcribed from linearized plasmids using a digoxigenin (DIG)-labeling mix (Roche Diagnostics, Mannheim, Germany) and SP6 or T7 RNA polymerase (Takara, Otsu, Japan), and digested with DNase I (Takara, Otsu, Japan). A total of 1 μg of the linearized plasmid DNA was used in a 20-μL reaction for DIG-RNA labeling. The probes were stored at −80 °C until use. 

LP FFPE samples were sectioned at 4 μm and mounted onto adhesive glass slides (Platinum Pro^®^; Matsunami Glass, Osaka, Japan). The sections were deparaffinized in xylene and rehydrated in a graded alcohol series, followed by incubation in ultra-pure water for 5 min. After washing with phosphate-buffered saline (PBS) with 0.1% Tween 20 (PBST), the sections were incubated in PBST including proteinase K (final concentration 0.01 mg/mL; Agilent Technologies Japan, Tokyo, Japan) for 5 min at 37 °C, and fixed in 4% paraformaldehyde (Nacalai Tesque, Kyoto, Japan) for 15 min at room temperature. After washing with PBST, pre-hybridization was performed in a hybridization buffer containing 50% deionized formamide, 5× saline-sodium citrate (SSC; 1× SSC = 0.15 M NaCl and 0.015 M sodium citrate, pH 7.0), 50 µg/mL heparin (Nacalai Tesque, Kyoto, Japan), 100 µg/mL *Escherichia coli* tRNA (Roche Diagnostics, Mannheim, Germany), 1% sodium dodecyl sulfate (SDS), and 0.1% Tween 20 at 60 °C for 1 h, and hybridization was performed with DIG-labeled RNA probes (1 µg/mL in hybridization solution) at 60 °C overnight in a humidified chamber. After hybridization, the sections were washed as follows: for 5 min in 50% formamide in 2× SSC with 0.1% Tween 20 at 60 °C; 5 min in 2× SSC at 60 °C; and 5 min in 0.2% SSC at room temperature. The sections were washed 3 times with PBST at room temperature, incubated with blocking buffer (0.5% blocking reagent in PBST, Roche Diagnostics, Mannheim, Germany) for 15 min, and incubated with 1:3000 anti-Digoxigenin-AP (alkaline phosphatase), Fab fragments (Roche Diagnostics, Mannheim, Germany) in the blocking buffer at 4 °C overnight. The sections were rinsed 3 times with PBST and washed with reaction buffer (100 mM Tris-HCl, 100 mM NaCl, 50 mM MgCl_2_, pH 8.0). Section detection was carried out using a nitroblue tetrazolium/5-bromo-4-chloro-3-indolyl phosphate solution (Roche Diagnostics, Mannheim, Germany) in the reaction buffer. The sections were washed 3 times with PBST and counterstained with Vector^®^ Nuclear Fast Red (Vector Laboratories, Burlingame, CA, USA). 

### 2.6. Double Fluorescence RNA-ISH

Double fluorescence RNA-ISH was performed using the TSA^TM^ Fluorescein System (PerkinElmer, Waltham, MA, USA) and TSA^TM^ Cyanine 5 System (PerkinElmer, Waltham, MA, USA). An anti-sense *E4* DIG-RNA probe was prepared as described above. Anti-sense *E5a* and *E5b* biotin probes were transcribed from linearized plasmid using Biotin RNA Labeling Mix (Roche Diagnostics, Mannheim, Germany) and SP6 or T7 RNA polymerase, and digested with DNase I as described above. The probes were stored at −80 °C until use. 

The process from sectioning to hybridization was the same as described above. Hybridization was performed with an anti-sense *E4* DIG-labeled RNA probe and anti-sense *E5a* biotin-labeled RNA probe (each 1 µg/mL in hybridization solution) or anti-sense *E4* DIG-labeled RNA probe and anti-sense *E5b* biotin-labeled RNA probe (each 1 µg/mL in hybridization solution) at 60 °C overnight in a humidified chamber. After hybridization, the sections were washed as follows: for 5 min in 50% formamide in 2× SSC with 0.1% tween at 60 °C; 5 min in 2× SSC at 60 °C; and 5 min in 0.2% SSC at room temperature. The sections were washed 3 times with PBST at room temperature, incubated with blocking buffer (0.5% blocking reagent in PBST) for 15 min, and incubated with 1:100 anti-Digoxigenin-POD (horse-radish peroxidase), Fab fragments (Sigma-Aldrich, Merck KGaA, Darmstadt, Germany) in the blocking buffer at 4 °C overnight. The sections were rinsed 3 times with PBST and washed with Tris-NaCl-Tween buffer (TNT buffer: 100 mM Tris-HCl, 150 mM NaCl, 0.1% Tween 20, pH 7.5). The fluorescein tyramide reagent from the TSA^TM^ Fluorescein System (PerkinElmer, Waltham, MA, USA) was diluted at 1:50 with the amplification diluent and mounted onto adhesive glass slides at room temperature for 10 min. Then, the sections were washed 3 times with PBST at room temperature. Endogenous peroxidases were blocked with 3% H_2_O_2_ in PBST at room temperature for 1 h. The sections were rinsed 3 times with PBST and incubated with Tris-NaCl-blocking buffer (TNB buffer: 0.5% blocking reagent in TNT buffer, TSA^TM^ Fluorescein System Kit). Streptavidin-HRP was diluted at 1:250 with TNB buffer (TSA^TM^ Cyanine 5 System; PerkinElmer, Waltham, MA, USA) and mounted on the slides for 30 min. The slides were washed 3 times with TNT buffer. The cyanine 5 tyramide reagent was applied to the slides for 10 min. Then, the slides were washed 3 times with TNT buffer and washed twice with PBS. Nuclei were counterstained with 10 µg/mL Hoechst 33,342 (Thermo Fisher Scientific, Tokyo, Japan) in PBST for 10 min. The slides were washed 3 times with PBS. Fluorescence was detected with an Axio Imager M2 microscope (Zeiss Japan, Tokyo, Japan) equipped with Colibri.2 light-emitting diode (LED) illumination and the Apotome.2 system (Zeiss Japan, Tokyo, Japan).

### 2.7. Immunohistochemistry for HPV-6 E4

#### 2.7.1. Preparation of an Anti-HPV-6 E4 Monoclonal Antibody

To generate a specific antibody against HPV-6 E1^E4, an alpaca variable domain of heavy chain of heavy-chain (VHH) antibody library was used as described previously [18]. Then, the alpaca *VHH* gene was fused with the mouse *IgG Fc* gene. The peptides and antibody were prepared by RePHAGEN Co., Ltd. (Okinawa, Japan).

#### 2.7.2. Evaluation of Antibody Specificity

*Plasmid Construction.* HPV-6 *E1^E4* and HPV-11 *E1^E4* were cloned into the pcDNA3.1+ vector, with the 3× FLAG-tag at the N-terminus [19]. Human Chemokine-like factor (CKLF)-like MARVEL transmembrane domain-containing 7 (*CMTM7*) tagged with 3× FLAG at the N-terminus was also used as described previously [19].

*Cell Culture and Transfection.* Human embryonic kidney HEK293T cells were cultured in high-glucose Dulbecco’s modified Eagle’s medium (DMEM) supplemented with 10% fetal bovine serum (FBS) and penicillin/streptomycin. HEK293T cells (2.0 × 10^6^ cells) were cultured in a 10-cm dish for 24 h. Immediately before transfection, the cells were rinsed and supplemented with fresh culture medium (high-glucose DMEM) without FBS and penicillin/streptomycin. Transfection was performed using HilyMax (Dojindo, Kumamoto, Japan). HPV-6 *E1*^*E4-*, HPV-11 *E1*^*E4-*, and human *CMTM7*-expressing plasmids (30 µg) were mixed with 15 µL HilyMax and 900 µL high-glucose DMEM and incubated for 15 min. Then, the DNA mixture was added to the cells. After 4-h incubation, high-glucose DMEM including 10% FBS and penicillin/streptomycin was added to the cells. The cells were cultured for 48 h after transfection. Non-transfected cells were included in the transfection experiments.

*Western Blotting for HPV-6 E4.* HEK293T cells expressing HPV-6 *E1*^*E4*, HPV-11 *E1*^*E4*, or human *CMTM7* and non-transfected HEK293T cells were lysed in a buffer containing 62.5 mM Tris-HCl (pH 6.8), 2% SDS, and 10% glycerol. Lysate protein concentration was measured using a DC Protein Assay Kit (Bio-Rad, Hercules, CA, USA). Each protein sample was prepared at 50 µg/10 µL in 62.5 mM Tris-HCl (pH 6.8), 2% SDS, 10% glycerol, 6% mercaptoethanol, and 0.01% bromophenol blue and boiled for 5 min. The samples were electrophoresed in 12.5% acrylamide SDS-PAGE and transferred to polyvinylidene difluoride membranes. To confirm the expression of HPV-6 E1^E4, HPV-11 *E1^E4*, and human CMTM7, FLAG protein was detected with an HRP-conjugated monoclonal anti-FLAG M2 antibody (Sigma-Aldrich, Merck KGaA, Darmstadt, Germany) diluted at 1:1000 with Can Get Signal Solution 2 (Toyobo, Osaka, Japan). To evaluate the specificity of the anti-HPV-6 E1^E4 antibody (alpaca VHH fused with mouse IgG1 Fc), the antibody was used as a primary antibody without dilution. A secondary antibody, goat IgG-conjugated with HRP against mouse IgG (H+L) (Bio-Rad, Hercules, CA, USA), was used at a dilution of 1:1000 with Can Get Signal Solution 2 (Toyobo, Osaka, Japan). Bands were visualized using Clarity Western ECL Substrate (Bio-Rad, Hercules, CA, USA) and the ChemiDoc XRS+ System (Bio-Rad, Hercules, CA, USA). 

#### 2.7.3. Immunohistochemistry for HPV-6 E1^E4

For HPV-6 *E1^E4* immunohistochemistry, 4-μm-thick sections from FFPE samples were deparaffinized in xylene and hydrated in a graded alcohol series. Epitope retrieval was achieved by heating at 100 °C for 10 min in 1 mM Ethylenediaminetetraacetic acid (EDTA) buffer (pH 8.0). Endogenous peroxidase activity was quenched by incubating the sections in 0.3% H_2_O_2_ in methanol for 20 min at room temperature. According to the manufacturer’s protocol, a SAB-PO Kit (Nichirei Bioscience, Tokyo, Japan) was used to detect immunoreactivity to HPV-6 *E1^E4*. After blocking non-specific reactions by incubation in 10% goat serum, the tissue slides were incubated with the primary antibody against HPV-6 *E4* (alpaca VHH fused with mouse IgG1 Fc) overnight at 4 °C. Subsequently, a biotin-labeled secondary antibody and peroxidase-labeled streptavidin were applied. Immunolabeling was visualized by incubation in 3-3′-diaminobenzidine, and stained slides were counterstained with hematoxylin.

### 2.8. Statistical Analysis

Viral mRNA levels (the proportion of viral mRNA expression as a percentage) were analyzed by the Kruskal–Wallis test, followed by the Dwass–Steel–Critchlow–Fligner multiple comparison test to detect statistically significant differences in the mRNA levels of the 9 viral genes. The correlations among the levels of the 9 viral mRNAs were analyzed by Spearman’s rank-order correlation test. The correlations between viral load and viral mRNA levels were analyzed as described above. Statistical significance was set at *p* < 0.05. Statistical analyses were performed using NCSS Statistical Software ver.12 (Kaysville, UT, USA).

### 2.9. Ethics Approval and Consent to Participate 

This study was approved by the Institutional Review Board of the University of Ryukyus and was carried out in accordance with the 1975 Declaration of Helsinki, as revised in 2008. Informed consent was obtained from all patients before enrollment.

## 3. Results

### 3.1. HPV DNA Distribution, Subtypes, and Viral Load in LP

The clinicopathological characteristics of 23 specimens obtained from 13 patients with HPV-6-infected LP are summarized in Table 1. The Derkay score [20] ranged from 2 to 17 (Table 1). In 21 specimens, the average viral load (mean ± standard error of the mean [SEM]) was 4.80 × 10^5^ ± 1.86 × 10^5^ copies/ng DNA (median, 2.85 × 10^4^, Table 2). Although there were not sufficient samples to analyze HPV subtype in 2 patients (cases 11 and 13), phylogenetic analysis revealed that patients 1, 7, and 11 were clustered within the HPV-6a subtype (JN252316) and patients 2 and 3 were categorized as the HPV-6b subgroup (X00203), whereas patients 4–6 and 8–10 were closely related to the HPV-6vc subtype (JN252316). There was no significant correlation between the clinical parameters (age, sex, tumor location, Derkay score, and HPV-6 subtype) and viral load/HPV mRNA expression levels.

**Figure 2:**
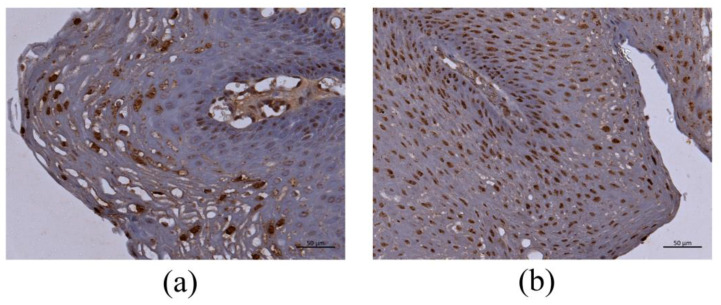
HPV-6-positive cells in LP by DNA-ISH. (**a**) The basal cells and middle to upper third layers of LP showed a positive reaction. Bar = 50 μm. (**b**) The positive reaction was observed in all LP layers. Bar = 50 μm.

The results of HPV DNA-ISH are shown in Table 3. HPV DNA-ISH generated positive signals in the nucleus and cytoplasm of the infected cells, although the intracellular distribution of the positive reaction pattern was different among the cases. Although the positive signals were distributed throughout LP (Figure 2a,b), there were more prominent reactions in the middle and upper cell layers (Figure 2a). 

### 3.2. Expression of HPV-6 mRNAs in LP

#### 3.2.1. Expression Levels of the 9 Viral mRNAs

**Figure 3:**
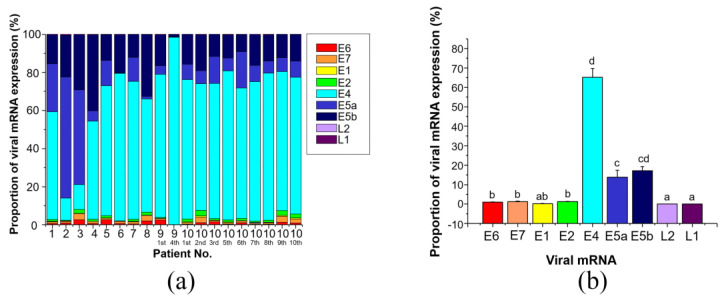
HPV-6 mRNA expression in individual cases and summation of all cases. (**a**) Viral mRNA expression in each case. There were some differences in the proportion of *E4*, *E5a*, and *E5b* expression. However, these 3 mRNAs were predominant compared with the other mRNAs examined. (**b**) Proportion of mRNA expression. *E4* was the most prevalent gene among the 9 viral mRNAs.

The expression levels of the 9 viral mRNAs were determined in 19 tissues from 10 patients (Table 2). *E4* mRNA expression (mean ± SEM) was the highest among these 9 genes (*E4*, 1152.94 ± 1012.18) and was followed by *E5b* and *E5a* mRNA levels (*E5b*, 25.78 ± 12.32; *E5a*, 11.13 ± 3.67). *E4* mRNA expression was the highest among the 9 viral genes in 8 of 10 patients, while *E5a* and *E5b* mRNA levels were higher than *E4* mRNA expression in patients 2 and 3 (Figure 3a). Meanwhile, the mRNA levels of *E6*, *E7*, *E1*, *E2*, *L2*, and *L1* were much lower than those of *E4*, *E5b*, and *E5a* (*E6*, 0.40 ± 0.05; *E7*, 0.94 ± 0.20; *E1*, 0.32 ± 0.09; *E2*, 1.23 ± 0.34; *L2*, 0.39 ± 0.30; *L1*, 0.12 ± 0.05). When the total expression of the 9 viral mRNAs was set to 100%, the percentage of *E4*, *E5a*, and *E5b* expression was 96% (*E4*, 65.12 ± 4.68%; *E5b*, 17.23 ± 1.98%; *E5a*, 13.76 ± 3.68% shown in Figure 3b), while that of the other 6 genes was only 4% (*E6*, 1.00 ± 0.19%; *E7*, 1.34 ± 0.21%; *E1*, 0.23 ± 0.05%; *E2*, 1.22 ± 0.17%; *L2*, 0.05 ± 0.01%; *L1*, 0.06 ± 0.02%). 

The correlations of the expression levels of each mRNA with the other viral mRNAs are shown in Table 4, examined by Spearman’s rank-order correlation. The levels of all 9 viral mRNAs were significantly correlated to the levels of the other mRNAs.

The correlations between viral DNA load and the levels of the 9 viral mRNAs were also examined by Spearman’s rank-order correlation (Table 5). Viral DNA load showed positive correlations with *E5a* and *L2* mRNA levels (*E5a*, r = 0.507, *p* = 0.0267; *L2*, r = 0.542, *p* = 0.0165), and also tended to show positive correlations with *E1* and *L1* mRNA levels, although these relationships did not reach statistical significance (*E1*, r = 0.449, *p* = 0.0536; *L1*, r = 0.436, *p* = 0.0619). In contrast, there was no correlation between viral DNA load and the mRNA levels of *E6*, *E7*, *E2*, *E4,* and *E5b* (r < 0.4, *p* > 0.05).

#### 3.2.2. Viral Load Alteration and Levels of the 9 Viral mRNAs in LP during 80 Months 

**Figure 4:**
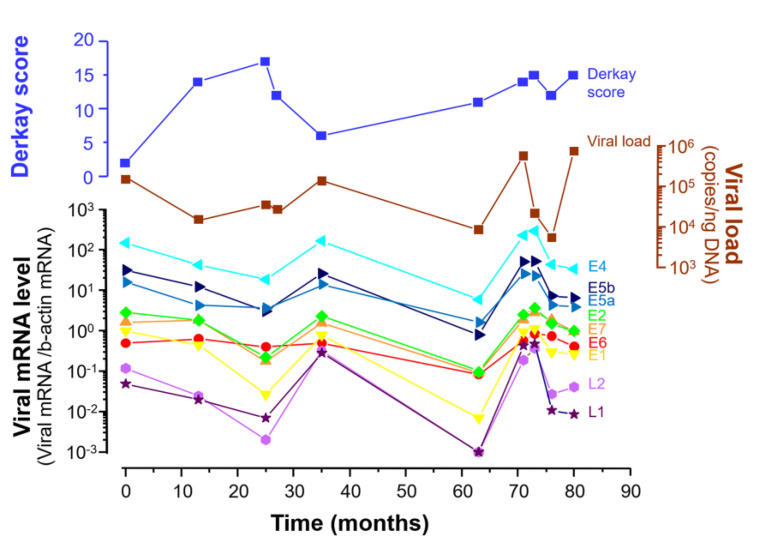
Alteration of mRNA levels, DNA viral load and Derkay score during long-term observation in patient 10. The viral DNA load alteration curve was similar to the mRNA expression curve.

Viral load and the expression of the 9 viral mRNAs were measured in case 10, who undertook 10 revision surgeries over a period of 80 months. The levels of the 9 viral mRNAs and viral load altered synchronously with each other’s peaks at 0, 35, and 71–73 months, with the lowest levels at 25 and 63 months (Figure 4). The shape of the mRNA expression curve resembled that of HPV-6 viral load. The Derkay score showed a different curve from viral mRNA levels and viral load.

#### 3.2.3. HPV-6 E6, E2, E4, E5a, and E5b mRNA Expression in LP Detected by RNA-ISH 

RNA-ISH with anti-sense probes showed that *E4*, *E5a*, *E5b*, *E6,* and *E2* signals were observed in 91.7% (11/12), 75% (9/12), 50.0% (6/12), 41.7% (5/12), and 41.7% (5/12) of LP cases, respectively (Table 3).

**Figure 5:**
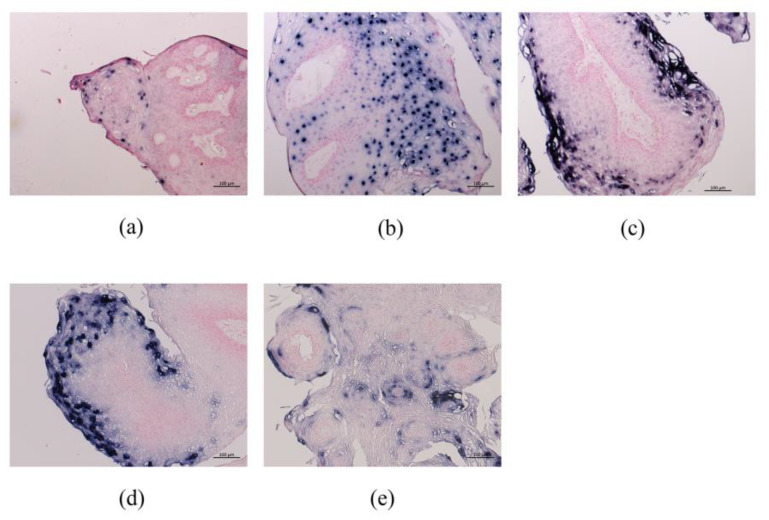
HPV mRNA expression in LP by RNA-ISH. Representative mRNA expression of (**a**) *E6*; (**b**) *E2*; (**c**) *E4*; (**d**) *E5a*; (**e**) *E5b*. *E4* and *E5a* were markedly expressed in the upper third of LP. Bar = 100 μm.

RNA-ISH demonstrated that *E4* and *E5a* mRNAs were strongly detected and located especially in the cytoplasm of the upper third cell layer of LP (Figure 5). Meanwhile, *E2* mRNA was expressed in the nuclei of the lower to upper third cell layer. *E5b* and *E6* mRNAs were sparsely expressed in the cytoplasm and nuclei of the upper third cell layer, respectively. 

**Figure 6:**
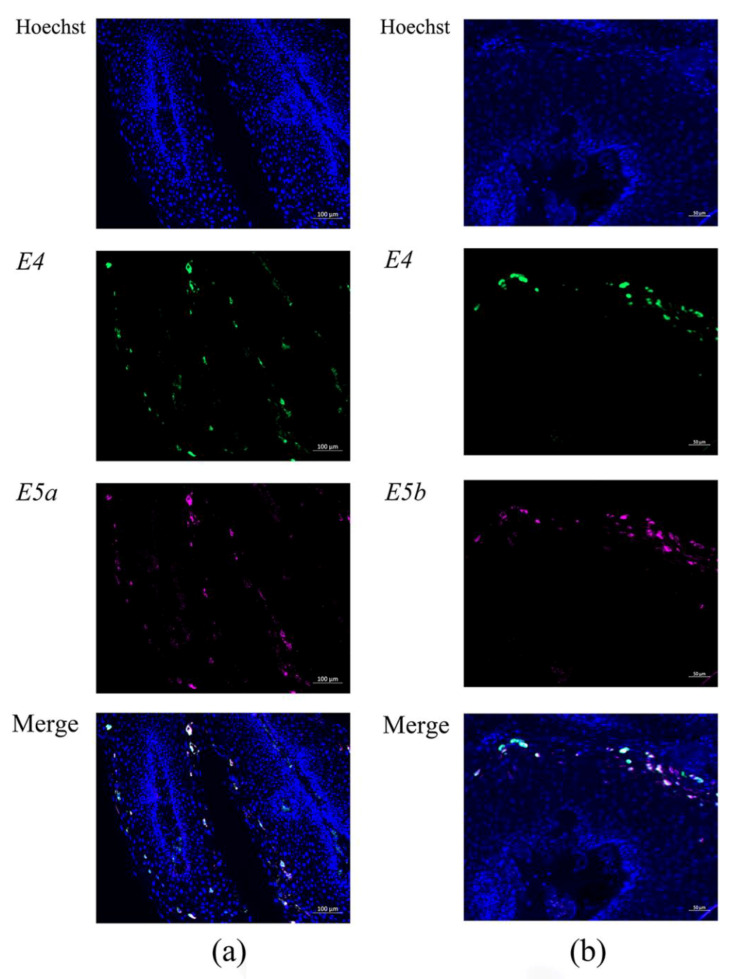
*E4* and *E5a*, and *E4* and *E5b* expression detected by double fluorescence RNA-ISH. (**a**) Double staining of *E4* and *E5a* in LP (bar = 100 μm). (**b**) *E4* and *E5b* in LP (bar = 50 μm). *E4* and *E5a* and *E4* and *E5b* mRNAs were co-expressed in the same LP cells.

Double fluorescence RNA-ISH showed that *E4* (green signal) and *E5a* (pink signal) mRNAs had a similar distribution pattern, and the merged image of *E4* and *E5a* showed that they were co-expressed in LP (Figure 6a). Double fluorescence RNA-ISH also showed that *E4* and *E5b* mRNAs had a similar distribution pattern, and the merged image of *E4* and *E5b* showed that they were also co-expressed in LP (Figure 6b). These *E4*, *E5a,* and *E5b* mRNAs were observed dominantly in the upper third cell layer of LP.

### 3.3. Immunohistochemistry for HPV-6 E1^E4

The newly developed monoclonal antibody against HPV-6 *E1^E4* was validated by western blot analysis. Western blot analysis revealed that the anti-HPV-6 *E1^E4* antibody bound only to HPV-6 E1^E4-3× FLAG fusion protein (13 kDa), but not to HPV-11 *E1^E4*–3× FLAG fusion protein, human CMTM7-3× FLAG fusion protein, or lysate proteins from HEK293T cells (Figure S1 in the Supplementary Materials). 

**Figure 7:**
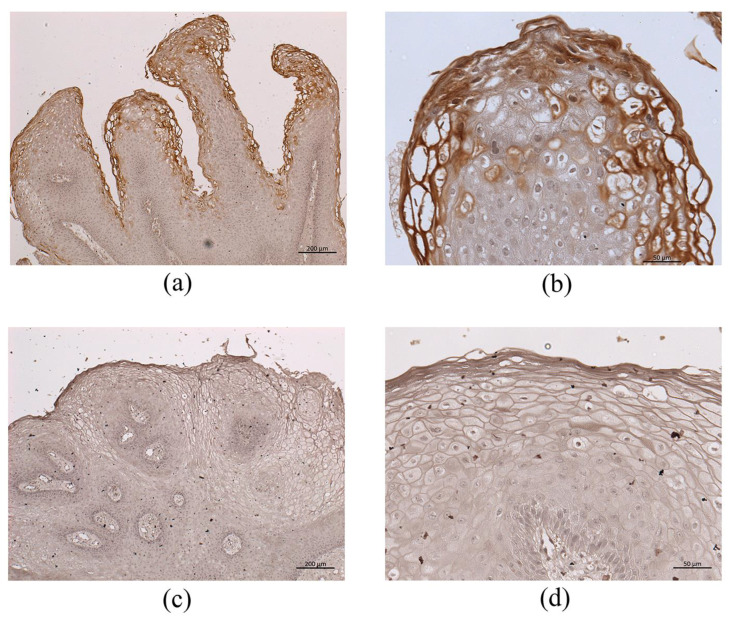
Immunohistochemistry for HPV-6 E4 in HPV-6-infected (**a**,**b**) and HPV-11-infected (**c**,**d**) LP. (**a**) Immunohistochemistry for HPV-6 E4 (low magnification) in HPV-6-infected LP (bar = 200 μm). (**b**) Immunohistochemistry for HPV-6 E4 (high magnification) in HPV-6-infected LP (bar = 50 μm). (**c**) Immunohistochemistry for HPV-6 E4 (low magnification) in HPV-11-infected LP (bar = 200 μm). (**d**) Immunohistochemistry for HPV-6 E4 (low high magnification) in HPV-11-infected LP (bar = 50 μm). Strong immunoreactivity was observed in (**a**,**b**), but there were no positive reactions in (**c**,**d**).

Immunohistochemistry for HPV-6 *E1^E4* generated a positive reaction in the upper third of LP cell layer, similar to *E4* RNA-ISH expression (Figure 7a,b). In contrast, HPV-11-infected LP did not show a positive reaction with this anti-HPV-6 *E1^E4* antibody (Figure 7c,d), but did show a positive reaction with a rabbit polyclonal anti-HPV-11 *E1^E4* antibody generated by ourselves (data not shown).

## 4. Discussion

According to a recent investigation of the global genomic diversity of HPV-6, HPV-6a belongs to sublineage B3, HPV-6b is lineage A, and HPV-6vc is sublineage B1 [21]. In 176 LP samples, 21.6% were HPV-6 lineage A, 47.2% were HPV-6 sublineage B1, and 17% were HPV-6 sublineage B3 [21]. In the present study, HPV-6 subtypes were successfully determined in 11 LP samples, showing 3 with HPV-6a, 2 with HPV-6b, and 6 with HPV-6vc. The distribution of HPV-6 subtypes in the present study is consistent with this previous report [21]. The transcriptional activity of the LCR is significantly higher in sublineage B1 with single nucleotide polymorphisms than in other B1 LCR variants [22]. Of the subtypes of HPV-6, HPV-6vc infection was observed in 2 cases with recurrence. Frequent recurrence of LP is a consequence of the long-term persistence of an identical initial HPV genomic variant [23]. Further study is needed to confirm the relationship between recurrence and HPV subtype or single nucleotide polymorphisms.

*E4*, *E5a*, and *E5b* mRNAs were exclusively expressed in the present study and represented 96% of the total expression of 9 HPV-6 mRNAs. The results suggest that these 3 genes are the most important for the HPV-6 life cycle. *E4* protein, which has significant sequence heterogeneity between HPV types, may play an essential role in the virus escape step from the epithelial surface and reflects the different tropisms and transmission routes of different papillomaviruses [10,24]. *E4*, *E5a*, and *E5b* were expressed in the middle to upper part of the epithelium and were co-expressed in the same cells, as observed using double fluorescence RNA-ISH. These results suggest that *E4*, *E5a*, and *E5b* work cooperatively for viral replication, virus release, and immunosurveillance avoidance. Although information for the DNA-ISH probe is not available, the DNA-ISH results in the present study showed that the cells expressing HPV-6 DNA were located in all LP layers, especially the middle to upper part of the tumor, in accordance with *E4*, *E5a,* and *E5b* mRNA expression. This observation also supports the above assumption related to *E4*, *E5a*, and *E5b*. Therefore, *E4*, *E5a*, and *E5b*, showing predominant expression in HPV-6-associated LP, are good candidates as therapeutic molecules in LP.

Recently, the raft culture method of LP was reported, and there was no significant relationship between *E1*, *E6*, *E7*, *L1*, and *L2* mRNA expression and viral load [25]. Although these results are inconsistent with our findings, the gene expression levels of *E1*, *E6*, *E7*, *L1*, and *L2* were much lower than those of *E4* and *E5* in the present study. These differences need to be clarified in the near future.

Previous studies regarding the functional roles of HPV-16 *E4* and *E5* reported that *E4* could destroy HPV-infected cells, and *E5* has a role in viral DNA synthesis and escape from immune surveillance [26]. HPV *E5* is a small, membrane-bound, highly hydrophobic protein, and the *E5* gene is conserved in many HPVs [27]. The function of *E5* has been investigated primarily in HPV-16, and it plays a vital role in the HPV life cycle by delaying normal epithelial cell differentiation, while maintaining cell cycle progression, enhancing the oncogenic ability of the significant transforming protein *E7* [14,27,28], and playing a role in immunoevasion [27,29]. However, the roles of *E5a* and *E5b* in HPV-6 remain unclear. Although the expression levels of the 9 viral mRNAs were correlated with each other in the present study (Table 4), viral DNA load was only correlated with *E5a* and *L2* mRNA levels (Table 5). L2 binds to circular viral DNA to enable genome encapsidation. The strong expression of *E5a* in the middle to upper part of LP suggests that *E5a* has a crucial role in viral DNA replication. 

In the present study, the long-term alteration of viral DNA load and mRNA expression were successfully measured in a patient with multiple instances of recurrence. The alteration of viral DNA load in patient 10 paralleled viral mRNA expression, and the expression of all mRNAs showed a similar alteration curve. These findings suggest that the expression of all mRNAs in human LP has a cooperative role in viral DNA synthesis to generate recurrent lesions.

An anti-HPV-6 *E4* antibody was established in the present study because *E4* had the highest expression among the 9 viral mRNAs examined in LP. This is the first antibody for HPV-6 *E4* that can be used for immunohistochemistry and Western blot analysis. The antibody did not react to any other HPV-related lesions, such as LP with HPV-11 infection and oropharyngeal carcinoma with HPV-16 infection (data not shown). In the high-risk types of HPV, *E4* protein assembles into amyloid fibrils that can disrupt the keratin network and compromise the normal assembly of the cornified envelope [10]. Although not yet defined precisely, *E4* amyloid fibers in low-risk HPV may contribute to virion release from the upper epithelial layers, and therefore affect infectivity and transmission [30]. *E4* immunohistochemistry demonstrated an extensively positive reaction in the upper cell layer in LP, in accordance with *E4* mRNA expression. These results suggest that E4 has a role in virus release from LP and contributes to viral transmission. All LP samples with HPV-6 infection showed a strong positive reaction in the upper cell layer. These findings suggest that the *E4* immunohistochemistry can be used for the detection of HPV-6 infection. Since the functional role of *E4* is not understood fully, this antibody will help in the analysis of neoplastic development and the viral life cycle in low-risk HPV.

## 5. Conclusions

In the present study, we examined the HPV DNA viral loads, expression levels of 9 viral mRNAs, and cellular distribution of mRNA expression in 13 LP patients with HPV-6 infection (3 with HPV-6a, 2 with HPV-6b, 6 with HPV-6vc, and 2 with undetermined type). *E4*, *E5a*, and *E5b* mRNAs accounted for 96% of the expression of the 9 mRNAs. The alteration of viral DNA load during recurrence paralleled the mRNA expression levels, and the expression of all mRNAs showed a similar curve. *E4*, *E5a*, and *E5b* were expressed in the middle to upper part of the epithelium and were co-expressed in the same cells. *E4* immunohistochemistry demonstrated an extensively positive reaction in the upper cell layer in accordance with *E4* mRNA expression. These results suggest that individual viral genes are coordinately expressed for viral replication, virus release, and immunosurveillance avoidance. However, there was no significant correlation between clinical parameters and viral load/HPV mRNA expression levels.

## Figures and Tables

**Table 1:** Clinical profiles of 13 patients with HPV-6-infected laryngeal papilloma (LP).

Case	Age (Years)	Sex	Surgery	No. of Tumors	Subsite	Derkay Score	HPV Subtype
1	27	M	3rd	Multiple	B, TVC; B, FVC	15	HPV-6a
2	43	M	1st	multiple	B, TVC; L, FVC	13	HPV-6b
3	34	M	2nd	multiple	B, FVC	6	HPV-6b
4	41	M	1st	multiple	B, TVC	4	HPV-6vc
5	41	M	1st	single	R, TVC	2	HPV-6vc
6	17	F	1st	multiple	B, TVC	6	HPV-6vc
7	28	M	1st	multiple	B, TVC	2	HPV6-a
8	4	F	1st	multiple	B, TVC	6	HPV-6vc
9	59	M	1st	multiple	B, TVC	6	HPV6-vc
	61		4th	multiple	B, TVC	3	
10	67	M	1st	multiple	B, TVC	2	HPV6-vc
	68		2nd	multiple	B, TVC	14	
	69		3rd	multiple	L, TVC	17	
	69		4th	multiple	B, TVC	12	
	71		5th	multiple	B, TVC	6	
	72		6th	multiple	B, TVC	11	
	73		7th	multiple	B, TVC	14	
	73		8th	multiple	B, TVC	15	
	73		9th	multiple	B, TVC	12	
	74		10th	multiple	B, TVC	15	
11	45	M	1st	multiple	B, TVC	6	HPV-6
12	44	M	1st	single	R, TVC	3	HPV-6a
13	44	M	1st	single	L, TVC	2	HPV-6

B, bilateral; F, female; FVC, false vocal cord; L, left; M, male; R, right; TVC, true vocal cord.

**Table 2:** Viral load and mRNA expression.

Case	Surgery	Viral Load (Copies/ng DNA)	HPV mRNA Expression/β-actin								
			*E6*	*E7*	*E1*	*E2*	*E4*	*E5a*	*E5b*	*L2*	*L1*
1	3rd	2,268,452	0.110	0.179	0.026	0.185	10.675	4.766	2.900	0.010	0.012
2	1st	649,319	0.335	0.312	0.105	0.159	4.496	24.633	8.496	0.062	0.134
3	2nd	436	0.134	0.163	0.010	0.109	0.677	2.585	1.513	0.002	0.006
4	1st	21,372	0.167	0.317	0.000	0.317	14.643	1.524	11.429	0.000	0.017
5	1st	25,347	0.425	0.153	0.010	0.213	11.474	2.241	2.309	0.001	0.002
6	1st	4739	0.135	0.218	0.003	0.059	16.934	0.004	4.460	0.000	0.010
7	1st	14,170	0.032	0.100	0.006	0.099	6.175	1.068	1.035	0.000	0.001
8	1st	36,408	0.394	0.554	0.018	0.322	11.688	0.278	6.403	0.002	0.004
9	1st	2,199,081	0.587	0.208	0.019	0.122	18.409	1.108	4.032	0.002	0.008
	4th	2,918,599	0.542	2.799	0.693	5.824	20,828.885	74.591	256.091	6.180	0.775
10	1st	166,859	0.501	1.627	0.991	2.838	149.595	16.359	32.002	0.118	0.048
	2nd	17,173	0.634	1.841	0.448	1.806	42.551	4.378	12.183	0.025	0.020
	3rd	39,002	0.401	0.177	0.027	0.223	18.655	3.723	3.061	0.002	0.007
	4th	28,595	NA	NA	NA	NA	NA	NA	NA	NA	NA
	5th	153,164	0.500	1.543	0.790	2.312	165.797	14.312	25.824	0.318	0.283
	6th	9551	0.083	0.092	0.007	0.103	5.951	1.657	0.796	0.001	0.001
	7th	645,354	0.566	1.893	0.946	2.516	231.081	26.454	51.181	0.190	0.423
	8th	24,404	0.849	2.830	1.132	3.716	290.630	23.532	52.166	0.373	0.477
	9th	6078	0.745	1.858	0.298	1.524	44.088	4.409	7.366	0.027	0.011
	10th	838,013	0.406	0.960	0.269	0.947	33.520	3.924	6.529	0.040	0.009
11	1st	NA	NA	NA	NA	NA	NA	NA	NA	NA	NA
12	1st	19,850	NA	NA	NA	NA	NA	NA	NA	NA	NA
13	1st	NA	NA	NA	NA	NA	NA	NA	NA	NA	NA

NA, not available.

**Table 3:** Summary of HPV DNA-ISH, mRNA-ISH, and E4 immunohistochemistry results.

Case	DNA-ISH			HPV RNA-ISH															E4-IHC		
				*E6*			*E2*			*E4*			*E5a*			*E5b*					
	L	M	U	L	M	U	L	M	U	L	M	U	L	M	U	L	M	U	L	M	U
1 *	−	1+	1+	NA	NA	NA	NA	NA	NA	NA	NA	NA	NA	NA	NA	NA	NA	NA	NA	NA	NA
2	1+	1+	3+	−	−	−	−	−	−	−	−	1+	−	2+	3+	−	−	−	−	1+	2+
3	3+	3+	3+	−	−	1+	−	1+	−	−	1+	1+	−	1+	2+	−	1+	2+	−	−	1+
4	3+	3+	3+	−	−	−	−	−	1+	−	−	1+	−	−	−	−	−	−	−	1+	1+
5	3+	3+	3+	−	−	−	−	−	−	−	−	−	−	−	−	−	−	−	−	1+	1+
6	−	2+	2+	−	−	−	−	−	1+	−	−	2+	−	−	1+	−	−	−	−	2+	2+
7	2+	2+	2+	−	−	−	−	−	−	−	−	2+	−	−	−	−	−	−	−	1+	2+
8	1+	3+	2+	−	−	−	−	−	−	−	1+	1+	−	−	1+	−	−	−	−	1+	1+
9	−	3+	3+	−	−	1+	−	−	1+	−	2+	3+	−	1+	2+	−	−	1+	−	2+	3+
10	−	3+	3+	−	−	1+	−	−	−	−	1+	2+	−	1+	2+	−	1+	2+	−	2+	3+
11	1+	2+	3+	−	2+	2+	2+	3+	2+	−	2+	3+	−	1+	1+	−	2+	3+	−	2+	3+
12	1+	1+	3+	−	−	−	−	−	1+	−	−	2+	−	2+	3+	−	−	1+	−	1+	2+
13	2+	3+	3+	−	1+	2+	−	−	−	−	1+	2+	−	1+	1+	−	1+	1+	−	1+	2+

ISH, in situ hybridization; IHC, immunohistochemistry; L, lower layer including the basal cell layer (lower third of the epithelium); M, middle layer; U, upper cell layer (upper third of the epithelium).−, negative expression; 1+, expression in less than 20% of cells; 2+, expression in 20–50% of cells; 3+, expression in more than 50% of cells.* The formalin-fixed paraffin-embedded (FFPE) sample of case 1 was not sufficient for evaluating RNA-ISH and E4-IHC.

**Table 4:** Correlation coefficients between the expression levels of the 9 viral mRNAs in HPV-6-related LP.

	*E6*	*E7*	*E1*	*E2*	*E4*	*E5a*	*E5b*	*L2*	*L1*
*E6*	-	0.786 ***	0.757 ***	0.754 ***	0.807 ***	0.498 *	0.705 ***	0.650 ***	0.577 ***
*E7*	0.786 ***	-	0.794 ***	0.882 ***	0.858 ***	0.649 **	0.942 ***	0.789 ***	0.838 ***
*E1*	0.757 ***	0.794 ***	-	0.853 ***	0.780 ***	0.845 ***	0.766 ***	0.944 ***	0.745 ***
*E2*	0.754 ***	0.882 ***	0.853 ***	-	0.861 ***	0.721 ***	0.872 ***	0.819 ***	0.743 ***
*E4*	0.807 ***	0.858 ***	0.780 ***	0.861 ***	-	0.554 ***	0.828 ***	0.705 ***	0.710 ***
*E5a*	0.498 *	0.649 **	0.845 ***	0.721 ***	0.554 ***	-	0.6965 ***	0.904 ***	0.814 ***
*E5b*	0.705 ***	0.942 ***	0.766 ***	0.872 ***	0.828 ***	0.6965 ***	-	0.781 ***	0.922 ***
*L2*	0.650 ***	0.789 ***	0.944 ***	0.819 ***	0.705 ***	0.904 ***	0.781 ***	-	0.806 ***
*L1*	0.577 ***	0.838 ***	0.745 ***	0.743 ***	0.710 ***	0.814 ***	0.922 ***	0.806 ***	-

* *p* < 0.05, ** *p* < 0.01, *** *p* < 0.001.

**Table 5:** Correlation coefficients between the levels of the 9 viral mRNAs and viral load in HPV-6-related LP.

mRNA	r	*p*-Value
*E6*	0.237	0.3289
*E7*	0.277	0.2506
*E1*	0.449	0.0536
*E2*	0.395	0.0944
*E4*	0.323	0.1777
*E5a*	0.507	0.0267 *
*E5b*	0.374	0.1150
*L2*	0.542	0.0165 *
*L1*	0.436	0.0619

* *p* < 0.05.

## Data Availability

All data generated or analyzed during this study are included in this published article.

## Supplementary Materials

The following are available online at https://www.mdpi.com/2076-2607/9/3/520/s1, Figure S1: SDS-PAGE gel of cell lines and western blot analysis using anti-E1^E4 antibody; Table S1: Primers used in the present study; Table S2: Target gene, method, standard DNA, detection range, and amplification efficiency in real-time polymerase chain reaction (PCR).
