# Supplementary material for: Coordinated Expression of HPV-6 Genes with Predominant E4 and E5 Expression in Laryngeal Papilloma
Source: Microorganisms. 2021 Mar 3;9(3):520. doi: 10.3390/microorganisms9030520 (PMC7998961; doi:10.3390/microorganisms9030520)
Supplement: Supplementary file 1 [file microorganisms-09-00520-s001.pdf]

Supplementary Materials for

Coordinated Expression of HPV-6 Genes with Predominant E4 and E5 Expression in Laryngeal Papilloma

Taro Ikegami, Hitoshi Hirakawa, Narutoshi Tsukahara, Akikazu Murakami, Norimoto Kise, Asanori Kiyuna, Takayoshi Kosugi, Shinya Agena, Hidetoshi Kinjyo, Narumi Hasegawa, Masatomo Touyama, Shunsuke Kondo, Hiroyuki Maeda, Mikio Suzuki, Akira Ganaha

Contents

Figure S1. SDS-PAGE of cell lines and western blot analysis using the anti-E1^E4 antibody.

Table S1. Primers used in the present study.

Table S2. Target gene, method, standard DNA, detection range, and amplification efficiency in real-time PCR.

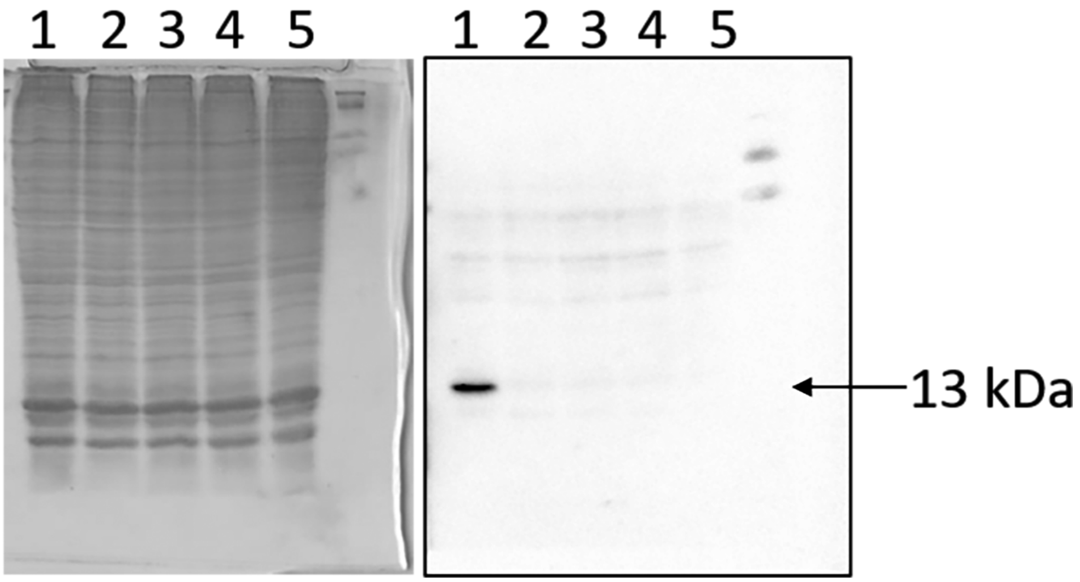

(a) (b)

Figure S1. SDS-PAGE of cell lines and western blot analysis using the anti-E1^E4 antibody. SDS-PAGE analysis with Coomassie brilliant blue staining. (b) Western blot analysis with the anti-E1^E4 antibody. Lane 1 = HEK293T cells transfected with pcDNA3.1+ HPV-6 E4-3× FLAG; Lane 2 = HEK293T cells transfected with pcDNA3.1+HPV-11 E4-3× FLAG; Lane 3 = HEK293T cells transfected with pcDNA3.1+ CMTM7-3× FLAG; Lane 4 = HEK293T cells transfected with pcDNA3.1+; Lane 5 = HEK293T cells. A protein with a molecular weight of 13 kDa (E4) was observed only in lane 1.

Table S1. Primers used in the present study.

**Screening primers**	**Sequence (5′–3′)**
GP5+	TTTGTTACTGTGGTAGATACTAC
GP6+	GAAAAATAAACTGTAAATCATATTC
MY09	CGTCCMARRGGAWACTGATC
MY11	GCMCAGGGWCATAAYAATGG
PC04	CAACTTCATCCACGTTCACC
GH20	GAAGAGCCAAGGACAGGTAC
LCR-F	ACGTAAGCGCGCCAAAAC
LCR-R	ATGCATATGAATAAATCTCTGCTGTG
**Cloning primers used for standard DNA**	**Sequence (5′–3′)**
F1	AAAGTGCAAATGCCTCCACG
R1	TTTCTCTACCTGCGTTCCCG
F2	TTATGGCTGCACGGTACGC
R2	TAACCCCAACACCCCTACAA
F3	GTGTGTTACTGTCCCGCTTG
R3	CACACCTAATGGCTGTCCCC
F4	GCAGGGGACAGCCATTAGG
R4	TCCACATGACGCATGTACTCT
**Real-time PCR primers and TaqMan probes**	**Sequence (5′–3′)**
E6-F	GCGTGCTGCCTAGAATTTCAT
E6-R	CAACAGTTGTTGCATATCCAGCAT
E6-Probe	FAM-CAAAGTGTCTATATTGGTTAATTTTTC-MGB
E7-F	GACGAAGTGGACGGACAAGA
E7-R	ACACTGCACAACCAGTCGAA
E1-F	TAAGTCCACGATTGGACGCC
E1-R	TTTCTCTACCTGCGTTCCCG
E2-F	GGCAAACACCACCTAAACGC
E2-R	CAGGAGTCAGTGTCCTGCAC
E4-F	CGAGGAGTCCAACAGTCACC
E4-R	TCTTTGGTGCTGGTCGTGAT
E5a-F	ATGGAAGTGGTACCTGTACAAATAGC

E5a-R	AGCAGTGTTAGTACTAGCACAGATG
E5b-F	GGCTGGGTTTGTGGTTGTTAT
E5b-R	ATTTGGTGTGTTTATCGCCTTG
L2-F	GTGCGTCAGCTACACAGCTA
L2-R	TGCCTATACCCAACCCTCCA
L1-F	ACCACACGCAGTACCAACAT
L1-R	TCCACATGACGCATGTACTCT
β-Globin-F	TGGGTTTCTGATAGGCACTGACT
β-Globin-R	AACAGCATCAGGAGTGGACAGAT
β-Globin-Probe	FAM-TCTACCCTTGGACCCAGAGGTTCTTTGAGT-TAMRA
β-Actin-F	GCGAGAAGATGACCCAGATC
β-Actin-R	CCAGTGGTACGGCCAGAGG
β-Actin-Probe	FAM-CCAGCCATGTACGTTGCTATCCAGGC-TAMRA
**Primers for ISH probes**	**Sequence (5′–3′)**
E6-ISH-F	CCACGTCTGCAACGACCATA
E6-ISH-R	TTGTCCAGCAGTGTAGGCAG
E2-ISH-F	ATGGAAGCAATAGCCAAGCG
E2-ISH-R	GGAGTCAGTGTCCTGCACATA
E4-ISH-F	GGGAAGTATGTTATGGCAGCAC
E4-ISH-R	TCTTTGGTGCTGGTCGTGAT
E5a-ISH-F	AGCTGCAGGAACAACCAGCA
E5a-ISH-R	GCTGTGTGTTCACAATGTAGTGG
E5b-ISH-F	ATGATGCTAACATGTCAATTTAATG
E5b-ISH-R	CTAATTCATATATATATAATCACCATCAGTAG
**Primers for pcDNA3.1(+)E1^E4 expression vector**	**Sequence (5′–3′)**
HPV-6 E1^E4-F<br>HPV-11 E1^E4-F	GAATTCACCACCATGGCGGACGATTCAGCAC
HPV-6 E1^E4-R	CTCGAGGCCACCGGATCCTAGGCGTAGCTGAACTGTTACTGT
HPV-11 E1^E4-R	CTCGAGGCCACCGGATCCTAGGCGTAGCTGCACTGTGA

R1 and R4 cloning primers used for standard DNA are the same as E1-R and L1-R real-time PCR primers, respectively. Since the HPV-6 sequence resembles that of HPV-11, HPV-6 E1^E4-F is the same as HPV-11 E1^E4-F.

Supplementary Table 2. Target gene, method, standard DNA, detection range, and amplification efficiency in real-time PCR.

**Target gene**	**Method**	**Standard DNA**	**Detection range**	**Amplification efficiency (%)**
E6	TaqMan MGB probe	p1478 HPV-6E6	10^1–10^7 copies	102.2
E7	SYBR Green	Clone A	2.0×10^1 – 2.0×10^7 copies	107.6
E1	SYBR Green	Clone A	2.0×10^1 – 2.0×10^7 copies	115.3
E2	SYBR Green	Clone B	2.0×10^1 – 2.0×10^7 copies	101.8
E4	SYBR Green	Clone B	2.0×10^1 – 2.0×10^7 copies	103.5
E5a	SYBR Green	Clone B	2.0×10^1 – 2.0×10^7 copies	102.0
E5b	SYBR Green	Clone C	2.0×10^1 – 2.0×10^7 copies	109.2
L2	SYBR Green	Clone C	2.0×10^1 – 2.0×10^7 copies	109.5
L1	SYBR Green	Clone D	2.0×10^1 – 2.0×10^7 copies	104.2

β-Actin	TaqMan probe	pCAG-mGFP-Actin	10^1–10^7 copies	108.2
β-Globin	TaqMan probe	Human placental DNA	0.3–300 ng	90.1
